# Vitamin D receptor (VDR) expression in different molecular subtypes of canine mammary carcinoma

**DOI:** 10.1186/s12917-021-02901-1

**Published:** 2021-05-25

**Authors:** R. Sánchez-Céspedes, M. D. Fernández-Martínez, A. I. Raya, C. Pineda, I. López, Y. Millán

**Affiliations:** 1 Department of Comparative Pathology, Veterinary Faculty, University of Córdoba, Carretera Madrid-Cádiz Km. 396, Campus Universitario de Rabanales, 14014 Córdoba, Spain; 2 Department of Medicine and Animal Surgery, Veterinary Faculty, University of Córdoba, Córdoba, Spain

**Keywords:** Dog, Canine mammary tumor, Comparative oncology, Immunohistochemistry, Vitamin D 3 receptor

## Abstract

**Background:**

The molecular-based classification of canine mammary carcinomas (CMCs) has been the focus of much current research. Both in canines and humans, the triple-negative (TN) molecular subtype of mammary cancer is defined by a lack of expression of progesterone receptor (PR), oestrogen receptor (ER) and HER2. It has a poor prognosis; no effective targeted therapy is available. Vitamin D displays anticarcinogenic properties, and the expression of its receptor (VDR) has been found in different molecular subtypes, being about 30–40 % of TN breast cancer (TNBC) positive to it. We assessed the VDR expression in the different molecular subtypes of 58 CMCs from 45 female dogs using an immunohistochemical panel for the molecular classification of included: PR, ER, HER2, cytokeratin (CK) 5, CK14, and Ki67. In addition, we studied the relationship among the molecular subtypes of CMCs and clinicopathologic parameters.

**Results:**

Investigation showed VDR positivity in 45.0 % of the triple-negative CMCs (TNCMCs), 27.3 % of luminal B and 19.0 % of luminal A. Luminal A was the most molecular subtype represented of the total tumours (36.2 %), followed of TNCMCs (34.5 %), luminal B (20.7 %) and HER2-overexpression (10.3 %). Both HER2-overexpression and TNCMC subtypes were positively related to lymphatic invasion (*P* = 0.028), simple histologic subtype (*P* = 0.007), a higher histological grade (*P* = 0.045) and a trend to higher proliferation index (*P* = 0.09).

**Conclusions:**

The highest VDR expression was observed in TNCMC, being almost half of them (45 %) positive to this receptor. VDR expression was absent in HER2-overexpression tumours and low in luminal A and B molecular subtypes.

**Supplementary Information:**

The online version contains supplementary material available at 10.1186/s12917-021-02901-1.

## Background

Historically, mammary gland tumours from both human and canine species have been classified based on an assessment of their histological type and grade. More recently, alternative molecular classifications introducing the gene expression profile have been proposed to redefine classification in human breast cancer (HBC), predict prognosis and guide therapy in routine clinical practice [1–3]. The gene expression profile is of limited utility in clinical practice, so immunohistochemistry (IHC) panels have been used widely as routine surrogates of the procedure in humans [4]. These IHC panels have been based on the expression profile of hormone receptors (oestrogen receptor, ER; progesterone receptor, PR), human epidermal growth factor receptor 2 (HER2), Ki67 and several basal markers [1, 3].

Canine mammary carcinomas (CMCs) are the most common neoplasm in female dogs and between 40 and 60 % of these tumours are malignant depending on the study [5]. The molecular classification of CMCs using IHC panels based on the human molecular classification has been a recent focus of research, but contradictory results have been produced, which may be due to the different methods of applying the criteria used in HBC classification [6–9].

Triple-negative (TN) breast cancer (TNBC), characterized by the loss of ER, PR and HER2 expression, represents about 15–25 % of breast cancer cases in women [10]. In female dogs, the percentage of this type of TN mammary cancer (triple-negative canine mammary carcinoma, TNCMC) amongst all CMCs varies widely between authors (18.7–76.3 %) [9, 11]. Both in humans and canines, TN mammary tumours (TNBC and TNCMC respectively) have a more aggressive phenotype, a higher rate of relapse and poorer prognosis than other mammary cancer types [9, 12–16]. Furthermore, TNBC is not treated with specific target therapies [13]; as such, it is important to explore novel therapeutic options for patients with this disease.

Calcitriol (1α, 25-dihydroxyvitamin D3; 1α,25-dihydroxycholecalciferol) is the active product of vitamin D synthesis and is regulated by a complex system of feedback mechanism mediated by enzymes, hormones and receptors [17]. The main function of calcitriol is to regulate calcium homeostasis, but many other biological effects are also exerted; most of them are mediated by the vitamin D receptor (VDR) signalling via, and VDR acts as a transcription factor of target genes [18].

Calcitriol/VDR influences many signalling pathways, including those regulating bone and calcium homeostatis and has also the capacity to regulate differentiation, proliferation and apoptosis of many cell types, and numerous cellular responses of the immune and cardiovascular systems (i.e. angiogenesis) [18–21].

The non-skeletal roles of vitamin D have been intensively investigated since the discovery that VDR was expressed by a wide range of cell types from human and canine tissues, including malignant cells [22–25]. It is expressed in most cancerous tissues, and it has been showed than calcitriol/VDR in cancer cells activates cyclin-dependent kinase inhibitors, inhibits mitogenic growth factors (EGF, IGF-1) and promotes the TGF-β activity, thus resulting in the inhibition of cell proliferation and cancer growth. Furhermore, calcitriol/VDR signalling has the capacity to downregulate cyclooxygenase-2, prostaglandin, and NF-kB pathways, thus inhibiting tumour-associated infammation, to suppress antiapoptotic proteins and to activate pro-apoptotic proteins. Thus, all previous mechanisms synergistically suppress tumour growth [16, 18, 21, 26].

VDR expression has been found in normal and neoplastic mammary glands from both humans [27–29] and canines by IHC techniques [30]. Due to its anticarcinogenic properties, vitamin D has emerged as a promising targeted therapy in cancer management, namely in HBC. To our knowledge, only one study has assessed VDR expression in all the different molecular subtypes of HBC [27]. However, in several studies, the expression of VDR in TNBC has been reported, indicating that about 27–40 % of TNBCs express VDR [27, 31, 32], and suggesting the possibility of using the VDR as a therapeutic target against TNBCs.

Some CMCs subtypes have been suggested as a valuable spontaneous cancer model to test new therapeutic strategies due to their similarities with some HBC subtypes in terms of epidemiology, pathology, genetic profile, and biological behaviour [33–37]. In dogs, only one study performed by our research group has been published about VDR expression in canine mammary glands [30], but it did not address the molecular subtypes of CMCs.

The main purpose of this study was to analyze VDR expression in all molecular subtypes of CMC. Furthermore, the second objective was to study the relationship among the molecular subtypes of CMC and their clinicopathologic characteristics.

## Results

### Characterisation of CMCs

The clinicopathological characteristics of the female dogs and their tumours are summarised in Table 1.

**Table 1:** Clinicopathologic parameters of the female dogs included in the study and their tumours

Variable	Range	No. of samples	Percent (%)
Age at diagnosis	<10 years	20	44.5
	≥10 years	25	55.5
Breed	Pure	22	48.9
	Crossing	23	51.1
No. of tumours	One	20	44.5
	More than one	25	55.5
Tumour size	<3 cm	43	74.1
	≥3 cm	15	25.9
Lymphatic invasion	Yes	16	27.6
	No	42	72.4
Histologic subtype	Simple	21	36.2
	Complex	27	46.6
	Mixed	10	17.2
Histologic grade	Grade 1	29	50.0
	Grade 2	18	31.0
	Grade 3	11	19.0
VDR expression	Positive cases	16	27.6
	Negative cases	42	72.4
Molecular subtype	Luminal A	21	36.2
	Luminal B	11	19.0
	HER2-overexpression	6	10.3
	Triple-negative	20	34.5
	Basal-like	18	31.0
	Non basal-like	*2*	3.4

According to the histologic subtype assessed by histology [haematoxylin and eosin (HE) stains], the complex was the more frequent subtype (27/58 cases, 46.6 %), followed by simple (21/58 cases, 36.2 %), and mixed (10/58 cases, 17.2 %). Tubulopapillary was the most common histotype of simple CMC (15/21 cases, 71.4 %), followed by solid carcinomas (6/21 cases, 28.6 %).

HER2, ER and PR expression was positive in luminal epithelial (LE) cells exclusively. Regarding HER2 score, 3/58 cases (5.2 %) were classified as 0, 14/58 cases (24.1 %) as 1, 24/58 cases (41.4 %) as 2 and 17/58 cases (29.3 %) were classified as 3. Taken into account that cases classified as IHC 3 + were considered HER2 positive, 17/58 cases (29.3 %) were HER2 positive and 41/58 cases (70.7 %) were HER2 negative. Data regarding ER and PR scores can be observed in Table 2.

**Table 2:** ER and PR scores

Score	ER (%)	PR (%)
0	8 (13.8)	4 (6.9)
1	0	0
2	23 (39.6)	24 (41.4)
3	1 (1.7)	3 (5.1)
4	5 (8.6)	5 (8.6)
5	8 (13.8)	6 (10.3)
6	6 (10.3)	10 (17.2)
7	5 (8.6)	3 (5,1)
8	2 (3.4)	3 (5.1)

Taken into account that a score of 3–8 was considered positive, 27/58 (45.6 %) and 30/58 cases (52.6 %) were ER and PR positive respectively.

A varied basal cytokeratins (CKs) expression pattern among histologic subtype of CMCs was observed. Thus, in most of complex CMCs (26/27 cases, 96.3 %) the neoplastic LE cells were positive to CK5, while in all cases (27/27 cases, 100 %) the residual or pre-existing non-proliferating ME cells observed at the periphery of the LE aggregates were CK5-positive, and in 7/27 cases (25.9 %) the proliferating neoplastic interstitial ME cells organized in fascicles or nests were CK5-positive. The CK14 expression in complex CMCs was observed in lower percentage of cases than CK5. Thus, in 10/27 cases (37.0 %) was observed ≥ 10 % of CK14-positive LE cells, while in all cases (27/27 cases, 100 %) the residual ME cells were CK14-positive, and in only 2/27 cases (7.4 %) were observed CK14-positive proliferating ME cells. Regarding the CKs expression in simple CMCs, in 18/21 cases (85.7 %) and in 11/21 cases (52.4 %) the proliferating LE cells were positive to CK5 and CK14 respectively. Furthermore, both CKs were observed in the flattened pre-existing non-proliferating ME cells in all cases (21/21 cases, 100 %) forming an irregular monolayer surrounding clusters of neoplastic LE cells. In mixed CMCs, the CK5 was observed in the LE and pre-existing ME cells of all cases (10/10 cases, 100 %), and in proliferating ME cells from 1/10 cases (10 %). Regarding the CK14 expression in mixed CMCs, it was observed in the pre-existing ME cells from all cases (10/10 cases, 100 %), in LE cells from 5/10 cases (50 %), and in the proliferating ME cells from 1/10 cases (10 %).

**Fig. 1:**
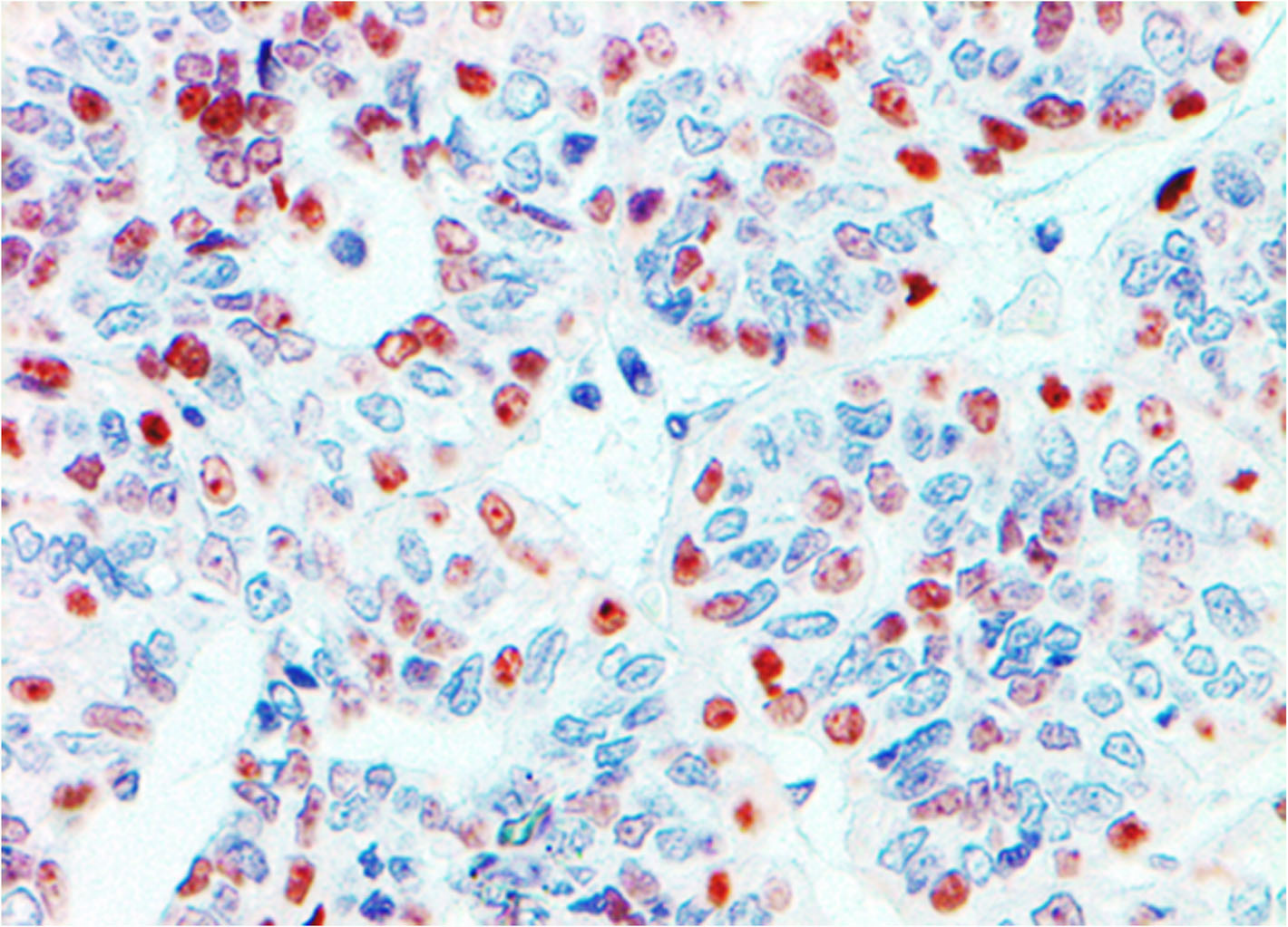
Immunohistochemistry for VDR of canine mammary carcinoma (Luminal B molecular subtype; complex histological subtype). The nuclei of the about the 30 % of tumour cells was moderate or strongly positive for VDR, so this case was diagnosed as VDR-positive. 40X

**Fig. 2:**
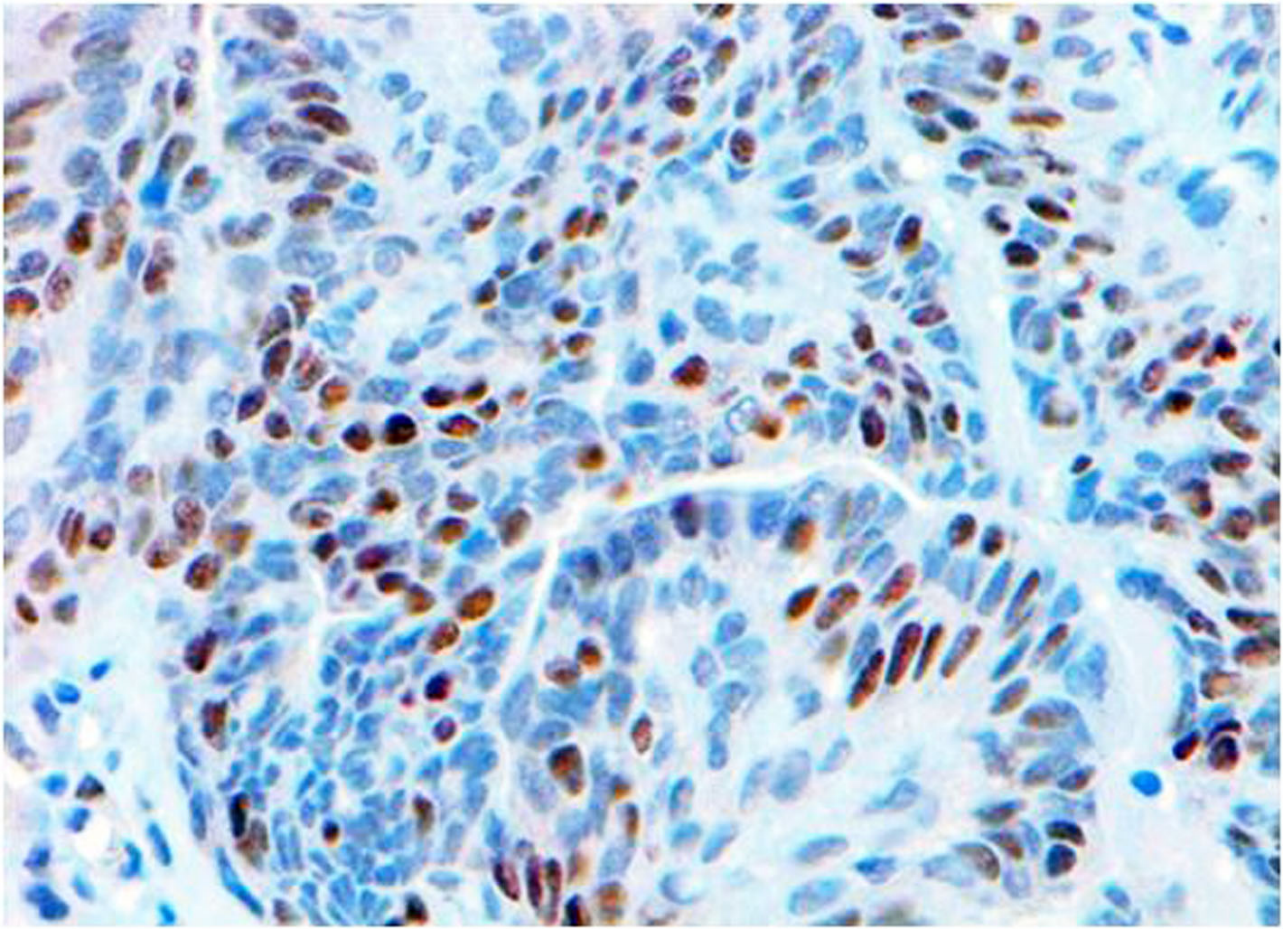
Immunohistochemistry for ER of canine mammary carcinoma (Luminal B molecular subtype; complex histological subtype). Most of tumour cells (about 30%) showed a strong immunostainingwith the anti-ER antibody in the nuclei, considering this case as ER-positive.40X

**Fig. 3:**
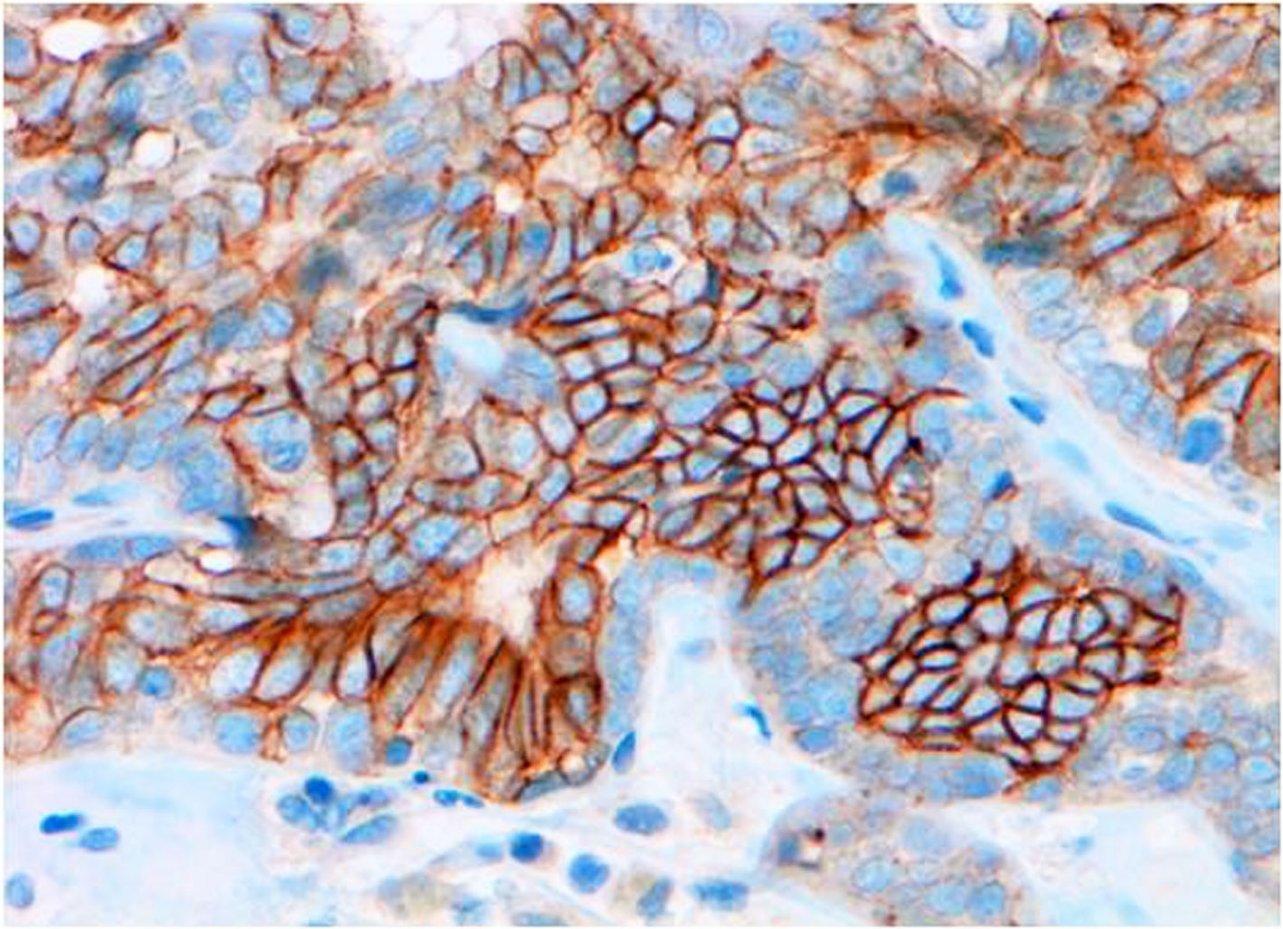
Immunohistochemistry for HER-2 of canine mammary carcinoma (Luminal B molecular subtype; complex histological subtype). More than 10% of tumour cells were found as HER2-positive cells with strong, complete, homogeneous membrane staining, considering the case as HER2-positive. 40X

**Fig. 4:**
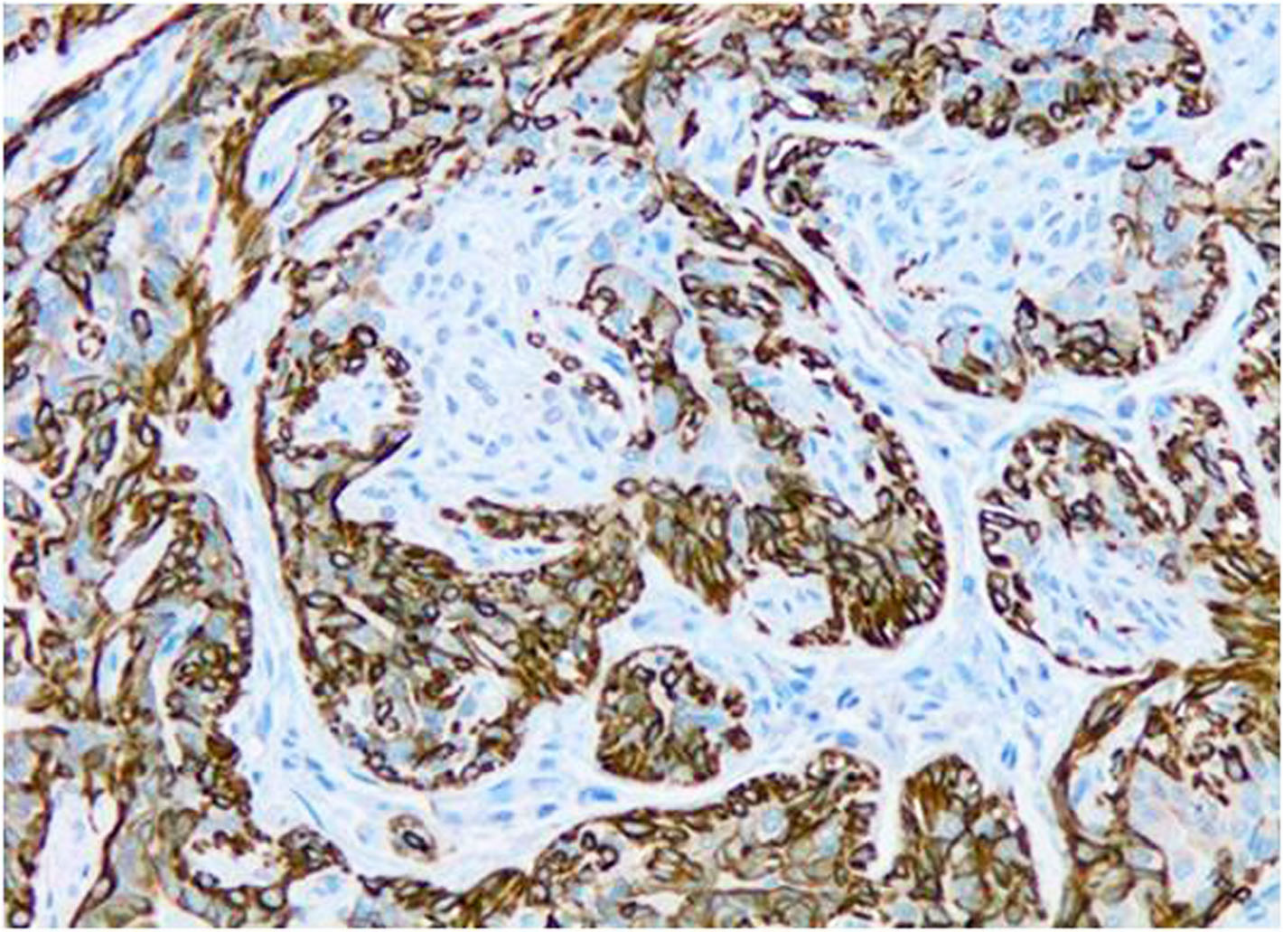
Immunohistochemistry for CK5 of canine mammary carcinoma (Luminal B molecular subtype; complex histological subtype). The image shows about 40% of neoplastic cells being CK5-positives with strong cytoplasmic staining. 20X

**Fig. 5:**
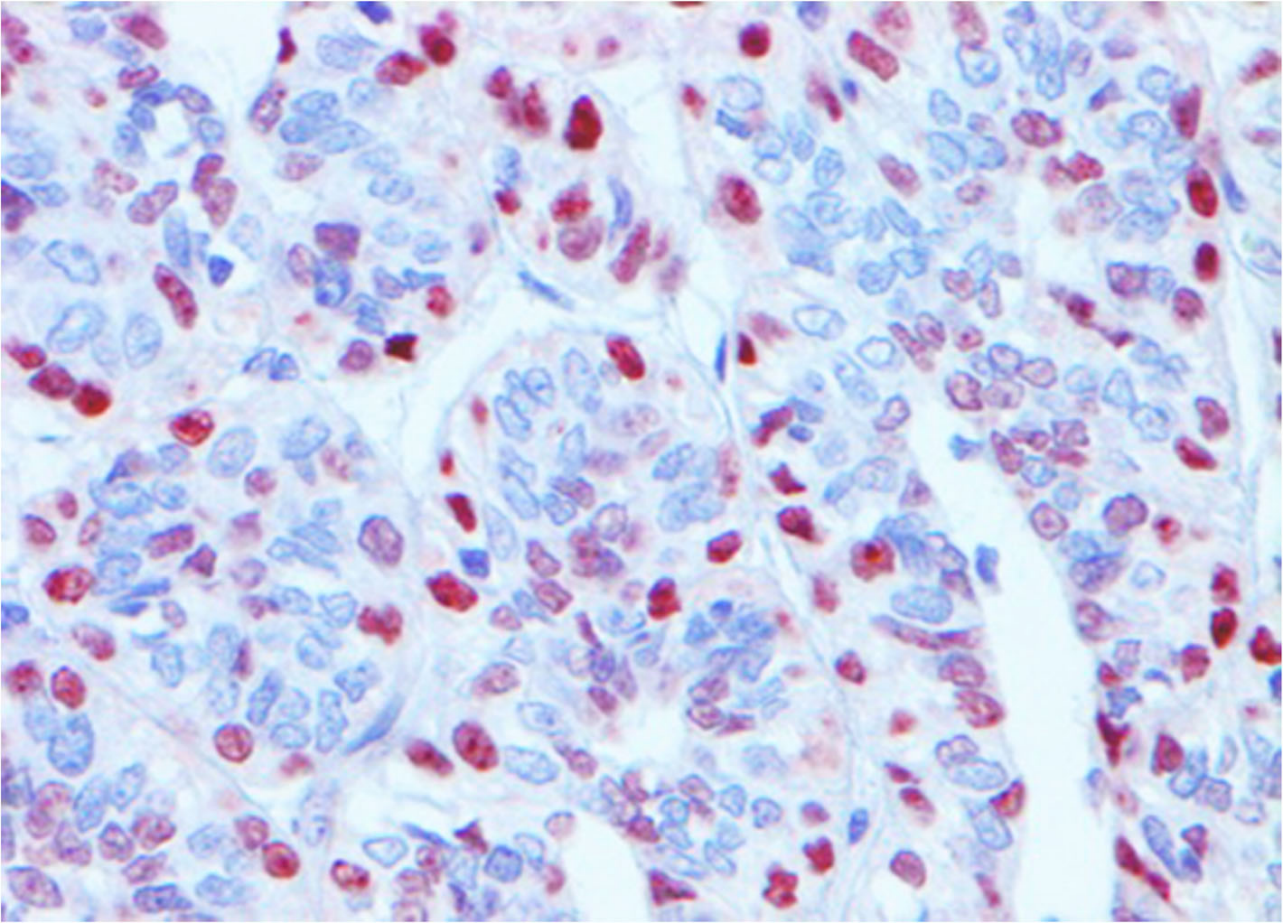
Immunohistochemistry for VDR of canine mammary carcinoma (TN basal-like molecular subtype; simple histological subtype)**. **The image shows VDR-positive oval to elongate cells morphologically indistinguishable from VDR-negative cells throughout the tumour. The staining intensity of the VDR-positive cells ranges from weak to strong. 40X

**Fig. 6:**
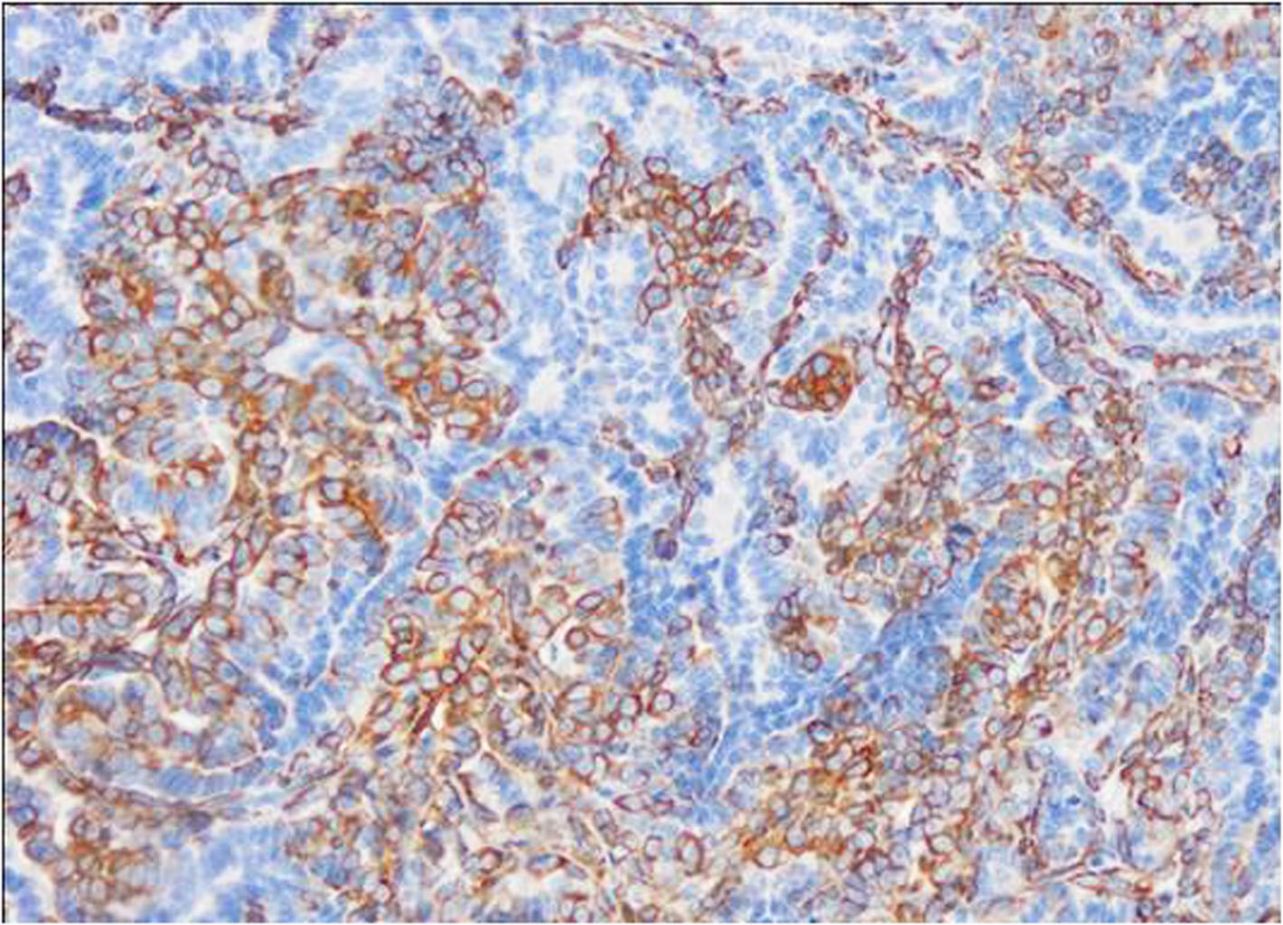
Immunohistochemistry for CK14 of canine mammary carcinoma (TN basal-like molecular subtype; simple histological subtype). The image shows CK14-positive cells representing about the 50% of the tumour cells. CK14-positive cells were morphologically indistinguishable from CK14-negative cells throughout the tumour. 20X

**Fig. 7:**
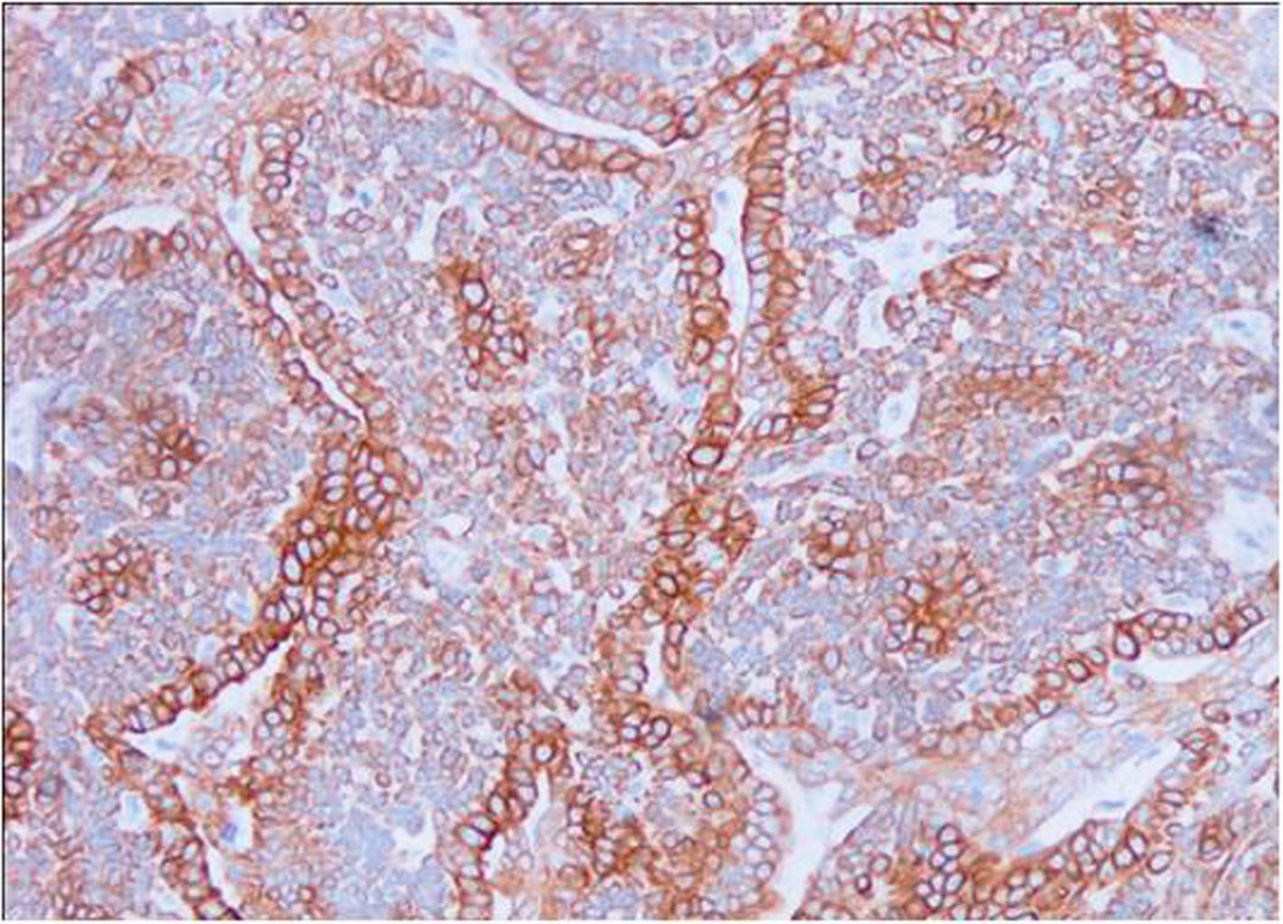
Immunohistochemistry for CK5 of canine mammary carcinoma (TN basal-like molecular subtype; simple histological subtype). The CK5 antigen was expressed in the most of tumour cells, although the intensity staining ranged from weak to strong. 20X

**Fig. 8:**
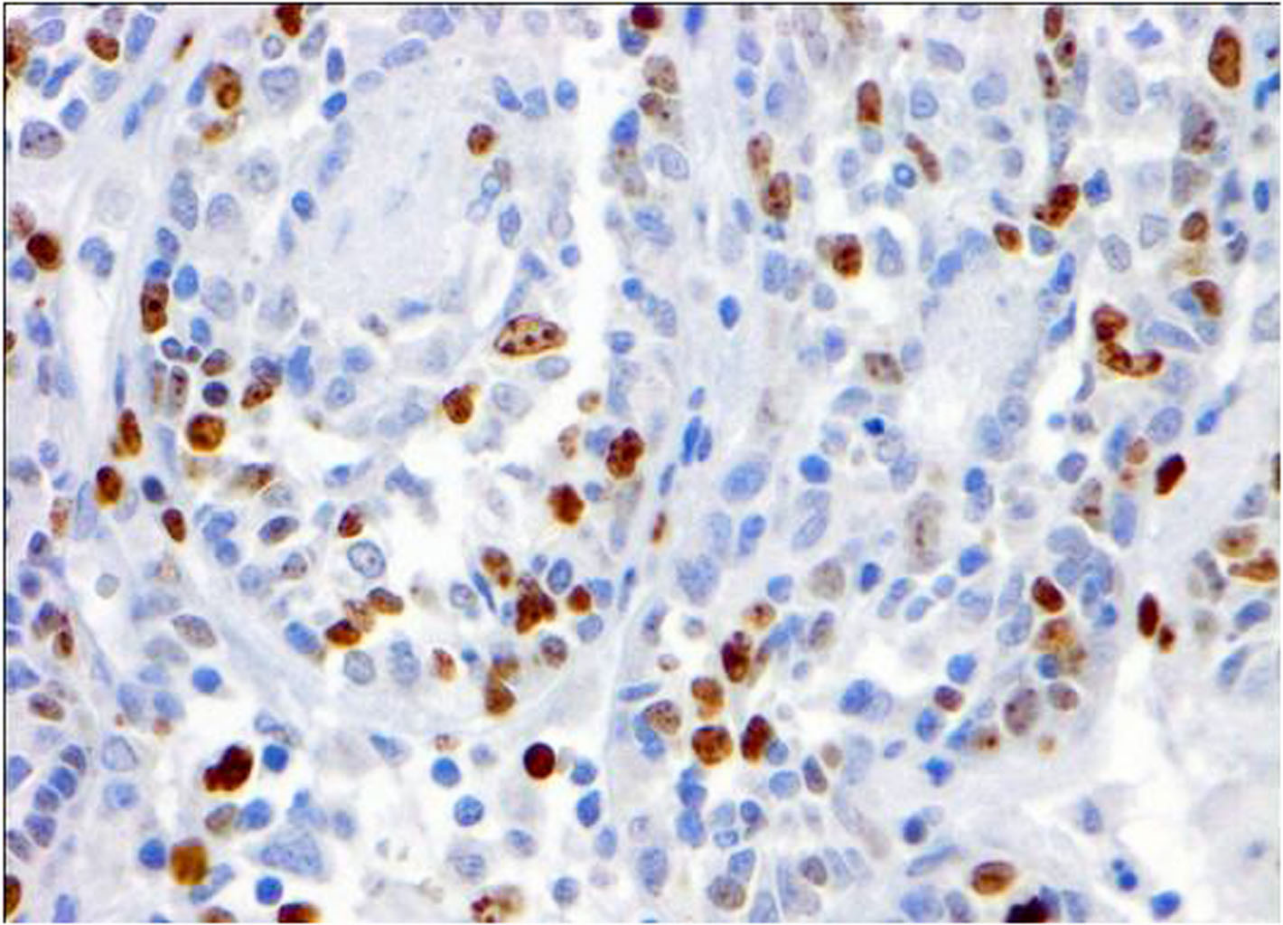
Immunohistochemistry for Ki67 of canine mammary carcinoma (TN basal-like molecular subtype; simple histological subtype). The image shows a high proliferation index, as determined for the high number of tumour cells (14.1%). 40X

According to the criteria adopted for the molecular classification of the CMCs, luminal A was the more frequent subtype (21/58 cases, 36.2 %), followed by TN (20/58 cases, 34.5 %), luminal B (11/58 cases, 19.0 %) (Figs. 1, 2, 3 and 4), and HER2-overexpression (6/58 cases, 10.3 %).TN subtype was divided in TN basal-like (18/58 cases, 31 %) (Figs. 5, 6, 7 and 8) and TN non basal-like (2/58 cases, 3.4 %) (Table 1). Moreover, an additional table shows the IHC results of the 58 tumors included in the study in more detail (see Additional file 1).

### Relationship between CMC immunophenotypes and clinicopathological characteristics

The relationship between the CMC immunophenotypes and clinicopathological characteristics was assessed (Table 3).

**Table 3:** Clinicopathologic characteristics in canine mammary carcinomas (CMCs) immunophenotypes

Variable	Range	No. of Luminal A cases (%)	No. of Luminal B cases (%)	No. of HER2+ cases (%)	No. of triple-negative cases (%)	*P* value
VDR expression	Positive	4 (19)	3 (27.3)	0	9 (45)	
	Negative	17 (81)	8 (72.7)	6 (100)	11 (55)	ns
ER expression	Positive	18 (85.7)	9 (81.8)	0	0	
	Negative	3 (14.3)	2 (18.2)	6 (100)	20 (100)	*P* < 0.001
PR expression	Positive	20 (95.2)	10 (90.9)	0	0	
	Negative	1 (4.8)	1 (9.1)	6 (100)	20 (100)	*P* < 0.001
HER2 expression	Positive	7 (33.3)	4 (36.4)	6 (100)	0	
	Negative	14 (66.7)	7 (63.6)	0	20 (100)	*P* = 0.002
Age	<10 y	11 (52.4)	6 (54.5)	1 (16.7)	9 (45)	
	≥10 y	10 (47.6)	5 (45.5)	5 (83.3)	11 (55)	ns
Breed	Pure	13 (61.9)	7 (63.6)	3 (50)	8 (40)	
	Crossing	8 (38.1)	4 (36.4)	3 (50)	12 (60)	ns
No. of Tumours	One	5 (23.8)	3 (23.7)	3 (50)	10 (50)	
	More	16 (76.2)	8 (72.7)	3 (50)	10 (50)	ns
Tumour size (cm)		2.1±0.8	2.3±0.9	3.1±0.4	1.9±0.3	ns
Lymphatic invasion	Yes	2 (9.5)	2 (18.2)	2 (33.3)	10 (50)	
	No	19 (90.5)	9 (81.8)	4 (66.7)	10 (50)	*P* = 0.028
Histologic subtype	Simple	7 (33.3)	1 (9.1)	5 (83.3)	8 (40)	
	Complex	13 (61.9)	4 (36.4)	1 (16.7)	9 (45)	
	Mixed	1 (4.8)	6(54.5)	0	3 (15)	*P* = 0.007
Histological grade	Grade 1	10 (47.6)	11 (100)	3 (50)	5 (25)	
	Grade 2	10 (47.6)	0	0	8 (40)	
	Grade 3	1 (4.8)	0	3 (50)	7 (35)	*P* = 0.045
IP		6.1 ± 2.9	22.2 ± 12.0	27.5 ± 18.1	16.7 ± 18.2	ns

*ns* not significant

Statistically significant differences were observed when comparing the CMC immunophenotypes and the ER, PR and HER2 expression, the lymphatic invasion, the histological subtype and the histological grade (Table 3).

The majority of luminal tumours (A and B) expressed both ER (18/21 cases, 85.7 %; and 9/11 cases, 81.8 % respectively) and PR (20/21 cases, 95.2 %; and 10/11 cases, 90.9 % respectively), while no HER2 + or TN tumours expressed these hormonal markers (*P* < 0.001, Chi-square test). Regarding the HER2 expression, all HER2-overexpression tumours were positive as expected, but less than half of luminal B (4/11 cases, 36.4 %) and luminal A (7/21 cases, 33.3 %) expressed HER2, and there was no HER2-positive TNCMC (*P* < 0.001, Chi-square test) (Table 3).

Ten out of 16 cases of lymphatic invasion (10/16 cases, 62.5 %) occurred in TN tumours, whereas 19/42 cases of non-lymphatic invasion (45.2 %) occurred in luminal A tumours (*P* = 0.028, Chi-square test). The histological subtype was associated with the molecular subtype (*P* = 0.007, Chi-square test). On the one hand, more than half of complex (17/27 cases, 63.0 %) and mixed (7/10 cases, 70 %) tumours according their histologic subtype had luminal immunophenotypes (A or B); on the other hand, more than half of simple tumours (13/21 cases, 61.9 %) were classified as HER2-overexpression or TN inmmunophenotypes (Table 3).

The histological grade was significantly associated with the molecular subtype (*P* = 0.0045, Chi-square test). All luminal B tumours (11/11 cases, 100 %) were classified as grade 1, while one half of the HER2-overexpression tumours (3/6 cases, 50.0 %) were classified as grade 1 and the other half as grade 3, and 10/21 luminal A cases (47.6 %) were classified as grade 1 and 2 at the same percentage. To note, whitin the group of grade 3 CMCs, 7/11 cases (63.6 %) were TN subtype (Table 3).

No statistically significant differences were found when comparing the CMC immunophenotype and the age of the dogs (< 10 years old vs. ≥10 years old), breed (pure vs. crossed), number of tumours (one vs. more than one), tumour size and PI (Table 3). However, it is interesting to indicate that although a statistically significant difference was not observed, a trend was found between the molecular subtype of CMC and IP (*P* = 0.09, Student´s t-test). HER2-overexpression subtype had the highest PI (27.5 ± 18.1 %), followed by the luminal B (22.2 ± 12.0 %), TNMCs (16.7 ± 18.2) (Fig. 8) and luminal A (6.1 ± 2.9) (Table 3). To note, the median value and range of all tumours was 15.0 ± 14.9.

### VDR expression in CMC immunophenotypes

VDR positivity was observed in 16/58 cases (27.6 %) (Table 1), and its immunoreactivity was observed in the nucleus of both LE and ME cells of the CMCs when the differentiation of these two cell types by morphology was possible. The distribution of the immunoreactivity varied among different histologic subtypes. VDR-positive cells in some CMCs were exclusively located around neoplastic epithelial tubules or solid nests or as trapped remnants and they appear to correspond to the pre-existing ME cells. Furthermore, in the cases of simple CMCs showing a solid pattern of growth (*n* = 6), VDR-positive cells were morphologically indistinguishable from VDR-negative cells within neoplastic nodules (Fig. 5). It is important to note that all 58 CMCs included in our study had some VDR staining (Figs. 1 and 5). Of the VDR-positive CMCs, 2/16 cases (12.5 %) had weak intensity, 6/16 cases (37.5 %) had moderate intensity (Fig. 1), and 8/16 cases (50.0 %) had strong intensity (Fig. 5). Regarding the percentage of VDR-positive cells in VDR-positive CMCs, 2/16 cases (12.5 %) were classified as 3 (21–30 % of positive cells), 2/16 cases (12.5 %) as 4 (31–40 % of positive cells), 4/16 cases (25.0 %) as 6 (51–60 % of positive cells), 4/16 cases (25.0 %) as 7 (61–70 % of positive cells), 2/16 (12.5 %) as 8 (71–80 % of positive cells), and 2/16 cases (12.5 %) as 9 (81–90 % of positive cells).

Although a statistically significant difference was not observed, a trend was found between the molecular subtype of CMC and VDR expression (*P* = 0.09, Chi-square test). Higher VDR expression was observed in TN tumours (9/20 cases, 45.0 %; Fig. 5), followed by luminal B (3/11 cases, 27.3 %) and luminal A (4/21 cases, 19.0 %; Fig. 1) (Table 3). When the TN subtype was subdivided according to the basal CK expression, 9/18 basal-like TN tumours (50.0 %) expressed VDR protein, whereas 2 non basal-like TN did not express it. All HER2-overexpression CMCs were VDR-negative (Table 3).

Taking into account the histological subtype of the CMC immunophenotypes, the results showed that 5/21 simple (23.8 %), 8/27 complex (29.6 %) and 3/10 mixed (30.0 %) CMCs expressed VDR protein (Table 4). To note, within the group of simple tumours that expressed VDR protein, the majority (4/5 cases, 80 %) were of TN molecular subtype.

**Table 4:** VDR expression in different immunophenotypes of canine mammary carcinomas (CMCs) according to their histological subtypes

Histological subtype (No.)	VDR expression (No.;%)	No. of Luminal A cases (%)	No. of Luminal B cases (%)	No. of HER2 + cases (%)	No. of triple-negative cases (%)	*P* value
Simple	Positive (5;23.8)	1 (14.3)	0	0	4 (50.0)	
(*n* = 21)	Negative (16;76.2)	6 (85.4)	1 (100)	5 (100)	4 (50.0)	ns
Complex	Positive (8;29.6)	3 (23.1)	1 (25)	0	4 (44.4)	
(*n* = 27)	Negative (19;70.4)	10 (76.9)	3 (75)	1 (100)	5 (55.6)	ns
Mixed	Positive (3;30)	0	2 (33.3)	0	1 (33.3)	
(*n* = 10)	Negative (7;70)	1 (100)	4 (66.7)	0	2 (66.7)	ns

*ns *not significant

## Discussion

The present study shows, to the best of our knowledge, the first investigation of VDR expression in the molecular subtypes of CMCs. Forty-five per cent of TNCMCs, 27.3 % of luminal B and 19.0 % of luminal A tumours expressed the VDR protein. A positive correlation was found between both the TN and HER2-overexpression molecular subtypes, and the lymphatic invasion, the simple histologic subtype, a higher histological grade and a trend to higher PI.

In our CMC cohort, we identified the luminal A, luminal B, HER2-overexpression and TN (subdivided into basal-like and non basal-like) subtypes. Other authors have also identified the molecular subtypes of CMCs, but a distinct panel of molecular markers and terminologies was used in each study, and there is not yet a consensus on this regard [6–9]. However, in the last work on this sense, Abadie et al. [9] concluded that CMCs, where all histological subtypes were included, are a valuable spontaneous models to test new therapeutic strategies for TNBC after used a panel of molecular markers proposed by Nielsen et al. [1] for HBC, as well as performed in our work.

Luminal A was the most represented subtype (36.2 %), closely followed by TNCMC (34.5 %). The high percentage of luminal A was similar to previous studies in canine species [6, 8], while the frequency of TNCMC ranges widely from 18.7 % [11] and 76.3 % [1], and may suggest that the protocols and the identification of the TN subtype are not yet well established in the dog. Moreover, this difference could be due to the inclusion criteria, due to the invasive nature of the CMCs was not consistently confirmed in some studies [6–8]. As such, the inclusion of *in situ* carcinomas in these studies could explain the relatively high frequency of the luminal A subtype. Furthermore, only in the study by Adabie et al. [9] and in the present report, was Ki67 used as a marker to differentiate luminal A from luminal B CMC subtypes.

In our study, when TNCMCs were subdivided according to their expression of basal CKs, most of them (18/20, 90.0 %) were basal-like TN (CK5 and/or CK14-positive expression), while the remaining few (2/20, 10.0 %) were non basal-like TN (CK-negative expression). Our results are in agreement with previous studies from both humans and canines, in which most TNBCs (about 80–90 %) express basal/myoepithelial CKs (the basal-like TN subtype) [9, 10, 16, 38].

Regarding the IHC expression of basal CKs, in our study was observed higher percentage of CK5-positive cases than CK14-positive cases (93.1 % *versus* 44.8 % respectively) regardless of the histological subtype, and the immunoreaction was observed mainly in neoplastic LE cells. In HBC, the CK5 was also more expressed than CK14, although the difference was lower than in CMCs (24.6 % *versus* 23.3 % respectively) [39]. Focusing on the proliferating ME cells, mean complex and mixed CMCs, CK5-positive expression was higher (25.9 and 10 %) than the CK14-positive expression (7.4 and 10 %), respectively. Thus, our results could be suggesting that proliferating ME cells in complex and mixed tumours were not terminally differentiated, according to previous studies in dog [40–43].

The HER2-overexpression subtype was diagnosed in a low percentage of the CMCs in our study (10.3 %), but it was diagnosed at a lower frequency by other authors [6, 8], and even not diagnosed by others [7, 9].This could be due to the recent controversy with regards to the IHC evaluation of the anti-HER2 antibody, since there are highly different expression rates in CMCs [38, 44, 45], or may be related to differences in antibodies, staining procedures and the evaluation system, although the main difference may have been the consideration of HER2-positive cells with different staining intensities (strong / moderate / weak to strong) [6, 9]. Furthermore, it is interesting to indicate that HER2-overexpression tumours with score 2 should underwent to *in situ* hybridization (FISH) for HER2 gene to confirm the expression or not of it.

We recently reported VDR protein expression in canine mammary tissue, with lower VDR expression in malignant tumours (26.5 %), followed by benign (40.0 %) and normal glands (100.0 %) [30].The present study corroborates our previous findings, since 27.6 % of CMCs expressed the VDR protein. A trend was observed in the relationship between higher VDR expression in the TN subtype (45.0 %) compared to the luminal B (27.3 %) and luminal A (19 %) subtypes.

This is the first study in which the expression of VDR protein in different molecular subtypes of CMC was analyzed, and it was interesting to note that a high percentage of TNCMC expressed this protein. Furthermore, focusing on the group of TNCMC of simple histological subtype in order to compare with HBC (mainly of an epithelial simple nature), half of them (50.0 %) expressed VDR. Our results are similar to those obtained in other studies in humans, in which about half of TNBCs express the VDR protein [26, 31].

In women, there is not an effective specific therapy against TNBC, and the possibility of the treatment with calcitriol or its analogues could be an alternative [31, 46]. However, only a few of the more than 1500 Vitamin D analogues have been tested *in vivo* so far [47, 48], so studies regarding VDR agonists with low calcemic effects are needed. Some authors have previously proposed different CMCs subtypes as a cancer model for some HBC subtypes [34–37, 40]. TNCMC of simple nature could be the best option since complex and mixed CMCs include a ME component that maybe could be conditioning the biological behaviour of tumours. Taken into account our results, TNCMC of simple histological seems to represent the subtype in which cancer management using vitamin D may have greater potential, since half of them express VDR, while the vast majority (85.4–100 %) of the other immunophenotypes are VDR-negative.

Each molecular subtype of CMC displayed significant distinctive pathological features. The HER2-overexpression and TN molecular subtypes were associated with established indicators of poor prognosis, such as lymphatic invasion, simple histologic subtype and high histologic grade. Furthermore, although not in a significant way (probably due to high standard deviation found), it was observed a trend to high PI. It could be indicative of intrinsic distinct biological characteristics, as reported in both humans and canines [6, 8, 9, 11, 28, 32, 38, 49].

The molecular subtype of CMC was related to the ER and PR expression, thus the majority of luminal tumours expressed both hormonal markers while HER2-overexpression or TN were absent to them as expect by their own definition. Furthermore, the molecular subtype was related to the histological subtype (simple vs. complex vs. mixed) in our study, in agreement other authors [6, 8]. In our series, more than half of complex and mixed CMCs were classified as luminal molecular subtype (A or B), which is in accordance with previous canine studies that reported that complex and mixed carcinomas are more likely to be ER positive [9, 50, 51]. Moreover, the ER expression has been reported to confer a positive influence on the prognosis in dogs [9, 50, 51] as well as in women [52, 53]. Eighty-three per cent (83.3 %) of the CMCs classified as HER2-overexpression and 40 % of the TN subtype were of the simple histological subtype; this is in accordance with other studies [6]. Simple CMCs have been shown to have a worse prognosis than complex and mixed CMCs [54].

In conclusion, TN was the molecular subtype of CMC most represented in our series (34.5 %), just behind luminal A (36.2 %), and the 45 % of them (TNCMC) expressed VDR protein according to IHC. In addition, TN subtype was related to lymphatic invasion, simple histologic subtype, a higher histological grade and it was a trend with higher PI. Furthermore, half (50 %) of TNCMC of simple nature expressed VDR protein, but the number of cases was low (*n* = 8), so further investigation should be performed to assess the VDR expression in CMC exclusively of simple histological type in order to assess the possible value of this entity as spontaneous animal model for TNBC expressing VDR protein.

## Methods

### Case selection

Forty-five bitches (45) were included in our study and a total of 77 tumours were recollected from the archives of the Department of Comparative Pathology of the University of Córdoba (Spain). Fifty-eight (58) mammary carcinomas were eligible for inclusion in the study when a histological diagnosis of invasive mammary carcinoma was established and confirmed by an incomplete layer of p-63 positive ME cells in order to differentiate *in situ* from invasive carcinomas [34, 55–57]. For this purpose, monoclonal mouse anti-p63 (clone 4A4), isotype IgG2 (Santa Cruz Biotechnology, Heidelberd, Germany), diluted 1:100, nuclear staining was used; this antibody was previously used in IHC studies in the dog [30, 58, 59]. Nineteen (19) tumours were excluded of our study because they were diagnosed as benign tumours (15) and as carcinoma *in situ* (4).

All samples were fixed in 10 % neutral buffered formalin for 24–72 h. After dehydration and embedding in paraffin wax, Sec. (3 μm) were cut from each block and stained with HE. All samples were classified in histological subtypes according to the presence of LE and/or ME and/or mesenchymal cells [3, 50] and the histologic grade of malignant tumours was also assessed by histology [59]. The 58 malignant tumours comprised 21 simple, 27 complex and 10 mixed type carcinomas. The simple CMCs were composed of a single population of columnar to cuboidal malignant LE cells disposed in a tubular, tubulopapillary or solid growth pattern. In the complex CMCs, the distinctive feature was the presence of spindle to stellate benign ME cells organized in interstitial bundles and/or whorls and admixed with the LE cells, the latter organized in tubules, papillae and/or nests. The mixed CMCs were diagnosed when benign cartilage/bone cells were also observed.

CMCs had been classified into histologic grade 1 (*n* = 29), grade 2 (*n* = 18) and grade 3 (*n* = 11).

### Immunohistochemistry

Seven different primary antibodies previously used in IHC studies in the dog were applied: (1) monoclonal rat anti-VDR [30]; (2) monoclonal mouse anti-human PR antibody [30, 44]; (3) polyclonal rabbit anti-ER [30]; (4) polyclonal rabbit anti-human c-erbB-2, [9, 34, 60, 61]; (5) monoclonal mouse anti-CK 5 [62]; (6) monoclonal mouse anti-human [63, 64]; and (7) monoclonal mouse anti-human Ki67 [9, 30, 42, 44, 64]. More detail about the primary antibody in Additional file 2. A commercial diluent (Dako, Burlingame, California) was used.

Tissue Sec. (4 μm thick) were placed on Vectabond-coated slides (Sigma Diagnostics, St Louis, Missouri). The slides were then deparaffined, rehydrated in a graded series of alcohol and incubated with 3 % hydrogen peroxide in methanol for 30 min. Heat-induced antigen retrieval was performed in a water bath at 96 ºC with 0.01 M citrate buffer (pH: 6.0) for 15 min for VDR, 25 min for PR, 40 min for ER, HER2 and Ki67, 10 min for CK14 and 30 min for CK5. After cooling for about 30 min at room temperature, sections were covered with 10 % normal goat serum in phosphate buffered saline for 30 min before incubation with one of the primary antibodies for 18 h at 4 ºC. Afterwards, the avidin-biotin-peroxidase complex method was applied, as recommended by the manufacturer (Vector Laboratories, Burlingame, California). Nova Red (Vector Laboratories, Burlingame, California) for the VDR antibody and 3,3-diaminobenzidine tetrahydrochloride (Sigma Diagnostics, St Louis, Missouri) for PR, ER, HER2, CK14, CK5 and Ki67 were used as chromogens. The slides were counterstained with Harris haematoxylin. As positive control tissues, canine duodenum tissue was used for VDR and Ki67, canine uterus tissue was used for PR and ER antibodies, human breast carcinoma tissue and CMC (simple of grade 3) expressing HER2 a score 3 were used for HER2 (control of technique and species control, respectively) and canine epithelial mammary tissue was used for CK14 and CK5. The normal mammary gland found in the 41 tissue sections under study was used as an internal positive control in every assay. As negative controls, the primary antibodies were replaced by rat IgG2b (Dako, Burlingame, CA, USA) for anti-VDR, mouse IgG2 (Dako, Burlingame, CA, USA) for anti-PR, immunoglobulin fraction of serum from non-immunised rabbits (Dako, Burlingame, CA, USA) for anti-ER and anti-HER2, and mouse IgG1 (Dako, Burlingame, CA, USA) for anti-CK14, anti-CK5 and Ki67 antibodies.

### Evaluation of IHC data

Each slide was evaluated independently by three veterinary pathologists (R.S.C, M.D.F.M, Y.M). When there was disagreement between evaluators (< 5 % of slides), cases were collectively reviewed using a multi-head microscope in order to achieve a consensus between the three observers.

VDR expression was evaluated in the same 10 random fields by the three pathologists through random images previously taken by one of them (R.S.C.) A semi-quantitative histological score based on the intensity and percentage of positive cells was used, regardless of their LE or ME nature [30]. Intensity was assessed as follows: 1 = weak, 2 = moderate and 3 = strong. The percentage of positive cells was assessed as follows: 1 = ≤ 10 %, 2 = 11–20 %, 3 = 21–30 %, 4 = 31–40 % and so on, to a maximum score of 10. The scores for intensity and percentage of positive cells were multiplied and the cases ranking from 5 to 30 were considered to be positive [30, 65]. To evaluate HER2 expression, we used the revised 2018 recommendation of the ASCO/CAP guidelines; 10 selected fields of the areas with the strongest protein expression were evaluated, and cases classified as IHC 3+ (complete membrane staining > 10 % of positive LE tumour cells) were considered HER2 positive [66].

PR and ER labelling were assessed using the Allred score [67], a semi-quantitative system that accounts for the staining intensity (scored on a scale of 0–3: 0 = none; 1 = weak; 2 = intermediate and 3 = strong) and the proportion of positive cells (scored on a scale of 0–5: 0 = none; 1=˂1 %; 2 = 1–10 %; 3 = 10–33 %; 4 = 33–66 % and 5 = 66–100 %), regardless of their ME or LE nature. Ten random fields were evaluated. The proportion and intensity were summed to produce total scores of 0 or 2 through 8. A score of 3 to 8 was considered positive.

For the IHC evaluation of CK5 and CK14 expression, both LE and ME (when presented, complex and mixed tumours) cells, in 10 random fields were evaluated, and cases with ≥ 10 % of positive tumour cells (it was not considered the residual/pre-existing ME cells) showing strong cytoplasmic staining were considered to be positive [41]. Ki67 expression was used to determine the proliferation index (PI). Images were captured (x40 microscope objective) from four fields with high number of Ki67-positive cells. The number of Ki67-positive and Ki67-negative LE cells was assessed by image analysis using ImageJ freeware. The PI was expressed as the percentage of positively-labelled cells. A minimum of 1000 tumour cells were counted per case [34].

### Grouping molecular subtypes

The 58 CMCs were grouped into molecular subtypes as follows: luminal A (ER + and/or PR+, “low” Ki67, any CK5 or CK14); luminal B (ER + and/or PR+, “high” Ki67, any CK5 or CK14); HER2-overexpression (ER-, PR-, HER2+, any CK5 or CK14); TN (ER- and PR-, HER2-). Moreover, TNCMCs were subdivided into basal-like TN (ER-, RP-, HER2-, CK5 and/or CK14+), and non basal-like TN (ER-, RP-, HER2-, CK5 and CK14-). Difference between luminal A and luminal B subtypes was the Ki67 expression (threshold at 14 %) [5].

### Statistical analyses

The statistical analyses were performed using SPSS software v.15.0. Kolmogorov-Smirnov test was used to evaluate data distribution. IHC and clinicopathological results were grouped into contingency tables and analyzed using a Fisher’s exact test. Relationships between discrete variables were performed using a Chi-square test. Student´s t-tests were employed to investigate associations between continuous and discrete variables. The correlation between continuous variables was studied using Pearson correlation coefficients. *P* ≤ 0.05 was considered statistically significant.

## Supplementary Information

**Additional file 1.** An additional table shows the immunohistochemical results of the 58 tumors included in the study in more detail.

**Additional file 2: **Table. Primary antibodies details.

## Data Availability

The datasets used and/or analysed during the current study are available from the corresponding author on reasonable request.

## References

[1] Nielsen TO, Hsu FD, Jensen K (2004). Immunohistochemical and clinical characterization of the basal-like subtype of invasive breast carcinoma. Clin Cancer Res.

[2] Rakha EA, Reis-Filho JS, Baehner F (2010). Breast cancer prognostic classification in the molecular era: the role of histological grade. Breast Cancer Res.

[3] Weigelt B, Reis-Filho JS (2010). Molecular profiling currently offers no more than tumour morphology and basic immunohistochemistry. Breast Cancer Res.

[4] Tang P, Skinner KA, Hicks DG (2009). Molecular classification of breast carcinomas by immunohistochemical analysis: are we ready?. Diagn Mol Pathol.

[5] Zappulli L, Peña L, Rasotto R, Goldschmidt MH, Gama A, Scruggs JL, Kiupel M, Kiupel M (2019). Volume 2: Mamamary Tumour. Surgical Pathology of Tumors of Domestic Animals.

[6] Gama A, Alves A, Schmitt F (2008). Identification of molecular phenotypes in canine mammary carcinomas with clinical implications: application of the human classification. Virchows Arch.

[7] Sassi F, Benazzi C, Castellani G, Sarli G (2010). Molecular-based tumour subtypes of canine mammary carcinomas assessed by immunohistochemistry. BMC Vet Res.

[8] Im KS, Kim NH, Lim HY, Kim HW, Shin JI, Sur JH (2014). Analysis of a new histological and molecular-based classification of canine mammary neoplasia. Vet Pathol.

[9] Abadie J, Nguyen F, Loussouarn D (2018). Canine invasive mammary carcinomas as models of human breast cancer. Part 2: immunophenotypes and prognostic significance. Breast Cancer Res Treat.

[10] Mills M, Yang GQ, Oliver DE (2018). Histologic heteroneneity of triple negative breast cancer: A National Cancer Centre Database analysis. Eur J Cancer.

[11] Kim NH, Lim HY, Im KS, Kim JH, Sur JH (2013). Identification of triple-negative and basal-like canine mammary carcinomas using four basal markers. J Comp Pathol.

[12] Lin NU, Vanderplas A, Hughes ME (2012). Clinicopathologic features, patterns of recurrence, and survival among women with triple-negative breast cancer in the National Comprehensive Cancer Network. Cancer.

[13] Engebraaten O, Vollan HKM, Borrensen-Dale AL (2013). Triple-negative breast cáncer and the need for new therapeutic targets. Am J Pathol.

[14] Schmadeka MD, Harmon BE, Singh M (2014). Triple-negative breast carcinoma. Am J Clin Pathol.

[15] Jaillardon L, Abadie J, Godard T (2015). The dog as a naturally-occurring model for insulin-like growth factor type 1 receptor-overexpressing breast cancer: an observational cohort study. BMC Cancer.

[16] Kim HW, Lim HY, Shin JI, Seung BJ, Ju JH, Sur JH (2016). Breed- and age-related differences in canine mammary tumors. Can J Vet Res.

[17] Dimitrov V, Salehi-Tabar R, An BS, White JH (2014). Non-classical mechanisms of transcriptional regulation by the vitamin D receptor: insights into calcium homeostasis, immune system regulation and cancer chemoprevention. J Steroid Biochem Mol Biol.

[18] Khammissa RAG, Fourie J, Motswaledi MH, Ballyram R, Lemmer J, Feller L (2018). The Biological Activities of Vitamin D and Its Receptor in Relation to Calcium and Bone Homeostasis, Cancer, Immune and Cardiovascular Systems, Skin Biology, and Oral Health. Biomed Res Int.

[19] Deeb KK, Trump DL, Johnson CS (2007). Vitamin D signalling pathways in cancer: potential for anticancer therapeutics. Nat Rev Cancer.

[20] Bandera Merchan B, Morcillo S, Martin-Nuñez G, Tinahones FJ, Macías-González M (2017). The role of vitamin D and VDR in carcinogenesis: Through epidemiology and basic sciences. J Steroid Biochem Mol Biol.

[21] Duffy MJ, Murray A, Synnott NC, O´Donovan N, Crown J (2017). Vitamin D analogues: Potential use in cancer treatment. Crit Rev Oncol Hematol.

[22] Gombart AF, Luong QT, Koeffler HP (2006). Vitamin D compounds: activity against microbes and cancer. Anticancer Res.

[23] Russell DS, Rassnick KM, Erb HN, Vaughan MM, McDonough SP (2010). An immunohistochemical study of Vitamin D receptor expression in canine cutaneous mast cell tumours. J Comp Pathol.

[24] Catwright JA, Gow AG, Milne E (2018). Vitamin D receptor expression in dogs. J Vet Intern Med.

[25] Davies J, Heeb H, Garimella R, Templeton K, Pinson D, Tawfik O. Vitamin d receptor, retinoid x receptor, ki-67, survivin, and ezrin expression in canine osteosarcoma. Vet Med Int. 2012;2:761034. 10.1155/2012/761034 PMC3544330 23346460

[26] Welsh J (2017). Function of the vitamin D endocrine system in mammary gland and breast cancer. Mol Cell Endocrinol.

[27] Al-Azhri J, Zhang Y, Bshara W (2017). Tumour expression of vitamin D receptor and breast cancer histopathological characteristics and prognosis. Clin Cancer Res.

[28] Huss L, Butt ST, Borgquist S (2019). Vitamin D receptor expression in invasive breast tumors and breast cancer survival. Breast Cancer Res.

[29] Francis I, AlAbdali N, Kapila K, John B, Al-Temaimi RA (2021). Vitamin D pathway related polymorphisms and vitamin D receptor expression in breast cancer. Int J Vitam Nutr Res.

[30] Sánchez-Céspedes R, Fernández-Martínez MD, Raya A, Pineda C, López I, Millán Y. Vitamin D receptor expression in canine mammary gland and relationship with clinicopathological parameters and progesterone/oestrogen receptors. Vet Comp Oncol. 2018;16(1):E185–93. 10.1111/vco.12371 29178579

[31] Thakkar A, Wang B, Picon-Ruiz M, Buchwald P, Ince TA (2016). Vitamin D and androgen receptor-targeted therapy for triple-negative breast cancer. Breast Cancer Res Treat.

[32] Soljic M, Mrklic I, Tomic S (2018). Prognostic value of vitamin D receptor and insulin-like growth factor receptor 1 expression in triple-negative breast cancer. J Clin Pathol.

[33] Ranieri G, Gadaleta CD, Patruno R (2013). A model of study for human cancer: spontaneous occurring tumors in dogs. Biological features and translation for new anticancer therapies. Crit Rev Oncol Hematol.

[34] Nguyen F, Peña L, Ibisch C (2018). Canine invasive mammary carcinomas as models of human breast cancer. Part 1: natural history and prognostic factors. Breast Cancer Res Treat.

[35] Abdelmegeed SM, Mohammed S (2018). Canine mammary tumors as a model for human disease. Oncol Lett.

[36] Zhang H, Pei S, Zhou B (2018). Establishment and characterization of a new triple-negative canine mammary cancer cell line. Tissue Cell.

[37] Gray M, Meehan J, Martínez-Pérez C (2020). Naturally-Occurring Canine Mammary Tumors as a Translational Model for Human Breast Cancer. Front Oncol.

[38] Perou CM, Sorlie T, Eisen MB (2000). Molecular portraits of human breast tumours. Nature.

[39] Engstrom MJ, Valla M, Bofin AM (2017). Basal markers and prognosis in luminal breast cancer. Breast Cancer Res Treat.

[40] Rasotto R, Goldschmidt MH, Castagnaro M, Carnier P, Calirai D, Zappulli V (2014). The dog as a natural animal model for study of the mammary myoepithelial basal cell lineage and its role in mammary carcinogenesis. J Comp Pathol..

[41] Sánchez-Céspedes R, Maniscalco L, Iussich S (2013). Isolation, purification, culture and characterization of myoepithelial cells from normal and neoplastic canine mammary glands using a magnetic-activated cell sorting separation system. Vet J.

[42] Sánchez-Céspedes R, Millán Y, Guil-Luna S, Reymundo C (2016). Espinosa de Los Monteros A, Martín de Las Mulas J. Myoepithelial cells in canine mammary tumours. Vet J.

[43] Sánchez-Céspedes R, Millán Y, Guil-Luna S (2018). Immunohistochemical and quantitative RT-PCR methods to assess RANK expression in normal and neoplastic canine mammary gland. J Vet Diagn Invest.

[44] Guil-Luna S, Sánchez-Céspedes R, Millán Y (2011). Aglepristone decreases proliferation in progesterone receptor-positive canine mammary carcinomas. J Vet Intern Med.

[45] Martín de las Mulas J, Millán Y, Dios R (2005). A prospective analysis of immunohistochemically determined estrogen receptor alpha and progesterone receptor expression and host and tumor factors as predictors of disease-free period in mammary tumors of the dog. Vet Pathol.

[46] Murray A, Madden SF, Synnott NC (2017). Vitamin D receptor as a target for breast cancer therapy. Endocr Relat Cancer.

[47] Jones G (2010). Vitamin D analogs. Endocrinol Metab Clin North Am.

[48] Mehta RG, Peng X, Alimirah F, Murillo G, Mehta R (2013). Vitamin D and breast cancer: emerging concepts. Cancer Lett.

[49] Fan C, Oh DS, Wessels L (2006). Concordance among gene-expression-based predictors for breast cancer. N Engl J Med.

[50] Goldschmidt M, Peña L, Rasotto R, Zappulli V (2011). Classification and grading of canine mammary tumours. Vet Pathol.

[51] Chang CC, Tsai MH, Liao JW, Chan JP, Wong ML, Chang SC (2009). Evaluation of hormone receptor expression for use in predicting survival of female dogs with malignant mammary gland tumours. J Am Vet Med Assoc.

[52] Iwase H, Zhang Z, Omoto Y, et al. Clinical significance of the expression of estrogen receptors alpha and beta for endocrine therapy of breast cancer. Cancer Chemother Pharmacol. 2003;52(Suppl 1):S34–8. 10.1007/s00280-003-0592-1 12819932

[53] Cheang MC, Chia SK, Voduc D (2009). Ki67 index, HER2 status, and prognosis of patients with luminal B breast cancer. J Nat Cancer Inst..

[54] Karayannopoulou M, Kaldrymidou E, Constantinidis TC (2005). Histological grading and prognosis in dogs with mammary carcinomas: application of a human grading method. J Comp Pathol..

[55] Pandey PR, Saidou J, Watabe K (2010). Role of myoepithelial cells in breast tumor progression. Front Biosci.

[56] Shamloula MM, El-Shorbagy SH, Saied EM. P63 and cytokeratin8/18 expression in breast, atypical ductal hyperplasia, ductal carcinoma in situ and invasive duct carcinoma. J Egypt Natl Canc Inst. 2007;202–2010 19190693

[57] Mardekian SK, Bombonati A, Palazzo JP (2010). Ductal carcinoma in situ of the breast: the importance of morphologic and molecular interactions. Human Pathol.

[58] Lopuszynski W, Szczubial M, Millán Y (2019). Immunohistochemical expresion of p63 protein and calponin in canine mammary tumours. Res Vet Sci.

[59] Peña L, De Andrés PJ, Clemente M, Cuesta P, Pérez-Alenza MD (2013). Prognostic value of histological grading in noninflamatory canine mammary carcinomas in a prospective study with two-year follow-up: relationship with clinical and histological characteristics. Vet Pathol.

[60] Burrai GP, Tanca A, De Miglio MR (2015). Investigation of HER2 expression in canine mammary tumors by antibody-based, transcriptomic and mass spectrometry analysis: is the dog a suitable animal model for human breast cancer?. Tumour Biol.

[61] Campos LC, Silva JO, Santos FS (2015). Prognostic significance of tissue and serum HER2 and MUC1 in canine mammary cancer. J Vet Diagn Invest.

[62] Ramalho LN, Ribeiro-Silva A, Cassali GD, Zucoloto S (2006). The expression of p63 and cytokeratin 5 in mixed tumors of the canine mammary gland provides new insights into the histogenesis of these neoplasms. Vet Pathol.

[63] Lai CL, van den Ham R, van Leenders G, van der Lugt J, Mol JA, Teske E (2008). Histopathological and immunohistochemical characterization of canine prostate cancer. Prostate.

[64] Kishimoto TE, Yoshimura H, Saito N (2015). Salivary gland epitelial-myoepithelial carcinoma with high-grade transformation in a dog. J Comp Pathol.

[65] Lopes N, Sousa B, Martins D (2010). Alterations in Vitamin D signalling and metabolic pathways in breast cancer progression: a study of VDR, CYP27B1 and CYP24A1 expression in benign and malignant breast lesions. BMC Cancer.

[66] Wolff AC, Hammond MEH, Allison KH (2018). Human epidermal growth factor receptor 2 testing in breast cancer: American Society of Clinical Oncology/College of American Pathologists Clinical Practice Guideline Focused Update. J Clin Oncol.

[67] Allred DC, Harvey JM, Berardo M, Clark GM (1998). Prognostic and predictive factors in breast cancer by immunohistochemical analysis. Mod Pathol.

