# Supplementary material for: Vitamin D receptor (VDR) expression in different molecular subtypes of canine mammary carcinoma
Source: BMC Vet Res. 2021 May 25;17:197. doi: 10.1186/s12917-021-02901-1 (PMC8152340; doi:10.1186/s12917-021-02901-1)
Supplement: Supplementary file 1 — Additional file 1. An additional table shows the immunohistochemical results of the 58 tumors included in the study in more detail. [file 12917_2021_2901_MOESM1_ESM.docx]

Table. Immunohistochemical results of the 58 tumors included in the study.

| Case | Molecular subtype | ER | | | | PR | | | | HER2 | | | | CK5 | | CK14 | |
| --- | --- | --- | --- | --- | --- | --- | --- | --- | --- | --- | --- | --- | --- | --- | --- | --- | --- |
|  |  | i score | % + cells score | Total score | +/- | i score | % + cells score | Total score | +/- | i | % + cells C/M | Total score | +/- | %  + cells | +/- | %  + cells | +/- |
| 1 | Basal-like TN | 1 | 1 | 2 | - | 1 | 1 | 2 | - | None | 0 | 0 | - | 30 | + | 15 | + |
| 2 | Luminal A | 2 | 2 | 4 | + | 3 | 3 | 6 | + | W | 5 | 1 | - | 20 | + | 5 | - |
| 3 | HER2-overexpres. | 0 | 0 | 0 | - | 0 | 0 | 0 | - | S | 20 | 3 | + | 50 | + | 5 | - |
| 4 | Luminal A | 3 | 1 | 4 | + | 4 | 2 | 6 | + | M | 15 | 2 | - | 40 | + | 5 | - |
| 5 | Basal-like TN | 0 | 0 | 0 | - | 1 | 1 | 2 | - | None | 0 | 0 | - | 60 | + | 15 | + |
| 6 | Luminal A | 5 | 3 | 8 | + | 5 | 3 | 8 | + | W | 5 | 1 | - | 25 | + | 5 | - |
| 7 | Luminal A | 4 | 3 | 7 | + | 3 | 3 | 6 | + | M | 15 | 2 | - | 45 | + | 35 | + |
| 8 | Luminal A | 5 | 2 | 7 | + | 2 | 2 | 4 | + | S | 15 | 3 | + | 30 | + | 20 | + |
| 9 | Basal-like TN | 1 | 1 | 2 | - | 1 | 1 | 2 | - | M | 30 | 2 | - | 15 | + | 5 | - |
| 10 | Luminal A | 3 | 1 | 4 | + | 2 | 1 | 3 | + | M | 5 | 1 | - | 40 | + | 20 | + |
| 11 | Basal-like TN | 0 | 0 | 0 | - | 1 | 1 | 2 | - | W | 5 | 1 | - | 20 | + | 10 | + |
| 12 | Basal-like TN | 1 | 1 | 2 | - | 1 | 1 | 2 | - | M | 15 | 2 | - | 30 | + | 5 | - |
| 13 | Basal-like TN | 1 | 1 | 2 | - | 1 | 1 | 2 | - | W | 5 | 1 | - | 45 | + | 5 | - |
| 14 | HER2-overexpres. | 0 | 0 | 0 | - | 1 | 1 | 2 | - | S | 15 | 3 | + | 20 | + | 5 | - |
| 15 | Non basal-like TN | 1 | 1 | 2 | - | 0 | 0 | 0 | - | M | 5 | 1 | - | 0 | - | 5 | - |
| 16 | Basal-like TN | 0 | 0 | 0 | - | 1 | 1 | 2 | - | W | 15 | 2 | - | 35 | + | 35 | + |
| 17 | Non basal-like TN | 0 | 0 | 0 | - | 1 | 1 | 2 | - | M | 5 | 1 | - | 5 | - | 5 | - |
| 18 | Luminal A | 1 | 1 | 2 | - | 3 | 2 | 5 | + | M | 15 | 2 | - | 5 | - | 5 | - |
| 19 | Basal-like TN | 1 | 1 | 2 | - | 1 | 1 | 2 | - | W | 5 | 1 | - | 5 | - | 5 | - |
| 20 | Luminal A | 1 | 1 | 2 | - | 2 | 1 | 3 | + | none | 0 | 0 | - | 30 | + | 20 | + |
| 21 | Luminal A | 5 | 3 | 8 | + | 5 | 3 | 8 | + | W | 40 | 2 | - | 40 | + | 20 | + |
| 22 | Luminal A | 1 | 1 | 2 | - | 4 | 2 | 6 | + | S | 20 | 3 | + | 20 | + | 5 | - |
| 23 | Basal-like TN | 1 | 1 | 2 | - | 1 | 1 | 2 | - | M | 20 | 2 | - | 65 | + | 30 | + |
| 24 | Basal-like TN | 1 | 1 | 2 | - | 0 | 0 | 0 | - | W | 5 | 1 | - | 20 | + | 15 | + |
| 25 | Luminal A | 3 | 2 | 5 | + | 3 | 2 | 5 | + | S | 30 | 2 | + | 70 | + | 30 | + |
| 26 | Luminal A | 3 | 2 | 5 | + | 2 | 1 | 3 | + | W | 5 | 1 | - | 20 | + | 30 | + |
| 27 | Luminal A | 3 | 3 | 6 | + | 5 | 3 | 8 | + | W | 15 | 2 | - | 50 | + | 5 | - |
| 28 | Luminal A | 5 | 2 | 7 | + | 1 | 1 | 2 | - | S | 15 | 3 | + | 25 | + | 5 | - |
| 29 | Luminal A | 3 | 2 | 5 | + | 3 | 2 | 5 | + | M | 20 | 2 | - | 30 | + | 40 | + |
| 30 | Luminal A | 4 | 3 | 7 | + | 4 | 2 | 6 | + | S | 5 | 2 | - | 40 | + | 5 | - |
| 31 | Basal-like TN | 1 | 1 | 2 | - | 1 | 1 | 2 | - | W | 15 | 2 | - | 40 | + | 30 | + |
| 32 | Basal-like TN | 1 | 1 | 2 | - | 1 | 1 | 2 | - | S | 5 | 2 | - | 30 | + | 5 | - |
| 33 | Luminal A | 4 | 3 | 7 | + | 4 | 2 | 6 | + | S | 15 | 3 | + | 80 | + | 50 | + |
| 34 | Luminal B | 3 | 2 | 5 | + | 1 | 1 | 2 | - | S | 20 | 3 | + | 40 | + | 30 | + |
| 35 | Basal-like TN | 1 | 1 | 2 | - | 1 | 1 | 2 | - | S | 5 | 2 | - | 80 | + | 30 | + |
| 36 | Luminal B | 4 | 2 | 6 | + | 3 | 2 | 5 | + | W | 15 | 2 | - | 50 | + | 20 | + |
| 37 | Luminal B | 2 | 1 | 3 | + | 2 | 2 | 4 | + | W | 5 | 1 | - | 30 | + | 5 | - |
| 38 | Luminal B | 3 | 2 | 5 | + | 4 | 2 | 6 | + | S | 20 | 3 | + | 20 | + | 5 | - |
| 39 | Luminal B | 4 | 2 | 6 | + | 4 | 3 | 7 | + | M | 20 | 2 | - | 40 | + | 5 | - |
| 40 | Luminal B | 2 | 2 | 4 | + | 3 | 3 | 6 | + | W | 5 | 1 | - | 10 | + | 5 | - |
| 41 | HER2-overexpress. | 0 | 0 | 0 | - | 1 | 1 | 2 | - | S | 30 | 3 | + | 15 | + | 20 | + |
| 42 | HER2-overexpres. | 1 | 1 | 2 | - | 0 | 0 | 0 | - | S | 15 | 3 | + | 30 | + | 5 | - |
| 43 | HER2-overexpres. | 1 | 1 | 2 | - | 1 | 1 | 2 | - | S | 15 | 3 | + | 20 | + | 5 | - |
| 44 | HER2-overexpres. | 0 | 0 | 0 | - | 1 | 1 | 2 | - | S | 20 | 3 | + | 10 | + | 5 | - |
| 45 | Luminal B | 1 | 1 | 2 | - | 2 | 2 | 4 | + | S | 15 | 3 | + | 20 | + | 5 | - |
| 46 | Luminal B | 3 | 3 | 6 | + | 4 | 3 | 7 | + | S | 5 | 2 | - | 15 | + | 20 | + |
| 47 | Basal-like TN | 1 | 1 | 2 | - | 1 | 1 | 2 | - | W | 15 | 2 | - | 35 | + | 5 | - |
| 48 | Luminal A | 2 | 3 | 5 | + | 2 | 2 | 4 | + | S | 15 | 3 | + | 30 | + | 5 | - |
| 49 | Luminal A | 3 | 3 | 6 | + | 4 | 3 | 7 | + | W | 5 | 1 | - | 10 | + | 5 | - |
| 50 | Basal-like TN | 1 | 1 | 2 | - | 1 | 1 | 2 | - | W | 15 | 2 | - | 30 | + | 20 | + |
| 51 | Luminal A | 2 | 2 | 4 | + | 2 | 2 | 4 | + | S | 5 | 2 | - | 40 | + | 30 | + |
| 52 | Luminal A | 3 | 2 | 5 | + | 4 | 2 | 6 | + | S | 15 | 3 | + | 20 | + | 5 | - |
| 53 | Basal-like TN | 1 | 1 | 2 | - | 1 | 1 | 2 | - | M | 20 | 2 | - | 10 | + | 5 | - |
| 54 | Basal-like TN | 0 | 0 | 0 | - | 1 | 1 | 2 | - | W | 5 | 1 | - | 20 | + | 20 | + |
| 55 | Basal-like TN | 1 | 1 | 2 | - | 1 | 1 | 2 | - | M | 15 | 2 | - | 30 | + | 20 | + |
| 56 | Luminal B | 3 | 3 | 6 | + | 3 | 2 | 5 | + | M | 40 | 2 | - | 10 | + | 5 | - |
| 57 | Luminal B | 1 | 1 | 2 | - | 2 | 3 | 5 | + | S | 20 | 3 | + | 40 | + | 5 | - |
| 58 | Luminal B | 3 | 3 | 6 | + | 3 | 3 | 6 | + | W | 15 | 2 | - | 30 | + | 20 | + |

i = intensity

TN = triple negative

CM = complete membrane

S = strong

W = weak

M = moderate
