# Supplementary material for: Vitamin D receptor (VDR) expression in different molecular subtypes of canine mammary carcinoma
Source: BMC Vet Res. 2021 May 25;17:197. doi: 10.1186/s12917-021-02901-1 (PMC8152340; doi:10.1186/s12917-021-02901-1)
Supplement: Supplementary file 2 — Additional file 2: Table. Primary antibodies details. [file 12917_2021_2901_MOESM2_ESM.docx]

Table. Primary antibodies details.

| Antibody | Clone | Isotype | Comercial | Dilution | Staining pattern |
| --- | --- | --- | --- | --- | --- |
| Monoclonal rat anti-VDR | 9A7 | IgG2b | Thermo Fisher Scientific, Rockford, Illinois | 1:100 | Nuclear |
| Monoclonal mouse anti-human PR antibody | 10A9 | IgG2 | Immunotech, Marseille, France | 1:500 | Nuclear |
| Polyclonal rabbit anti-ER | - | - | Zymed Laboratories, San Francisco, California | 1:200 | Nuclear |
| Polyclonal rabbit anti-human c-erbB-2 | - | - | Dako, Glostrup, Denmark | 1:200 | Membranous |
| Monoclonal mouse anti-CK 5 | XM26 | IgG1 | Leica, Newcastle, United Kingdom | 1:150 | Cytplasmic |
| Monoclonal mouse anti-human CK14 | clone LL002 | IgG1 | Biogenex, Molenstraat, Netherlands | 1:40 | Cytplasmic |
| Monoclonal mouse anti-human Ki67 | MIB-1 | IgG1 | Dako, Glostrup, Denmark | 1:75 | Nuclear |
